# Biomod2 modeling for predicting the potential ecological distribution of three *Fritillaria* species under climate change

**DOI:** 10.1038/s41598-023-45887-6

**Published:** 2023-11-01

**Authors:** Deya Huang, Qiuju An, Sipei Huang, Guodong Tan, Huige Quan, Yineng Chen, Jiayu Zhou, Hai Liao

**Affiliations:** School of Life Science and Engineering, Southwest Jiaotong University, Chengdu, 610031 Sichuan China

**Keywords:** Ecology, Ecology

## Abstract

The *Fritillaria* species ranked as a well-known traditional medicine in China and has become rare due to excessive harvesting. To find reasonable strategy for conservation and cultivation, identification of new ecological distribution of *Fritillaria* species together with prediction of those responses to climate change are necessary. In terms of current occurrence records and bioclimatic variables, the suitable habitats for *Fritillaria delavayi*, *Fritillaria taipaiensis*, and *Fritillaria wabuensis* were predicted. In comparison with Maxent and GARP, Biomod2 obtained the best AUC, KAPPA and TSS values of larger than 0.926 and was chosen to construct model. Temperature seasonality was indicated to put the greatest influence on *Fritillaria taipaiensis* and *Fritillaria wabuensis*, while isothermality was of most importance for *Fritillaria delavayi*. The current suitable areas for three *Fritillaria* species were distributed in south-west China, accounting for approximately 17.72%, 23.06% and 20.60% of China's total area, respectively. During 2021–2100 period, the suitable habitats of *F. delavayi* and *F. wabuensis* reached the maximum under SSP585 scenario, while that of *F. taipaiensis* reached the maximum under SSP126 scenario. The high niche overlap among three *Fritillaria* species showed correlation with the chemical composition (*P* ≤ 0.05), while no correlation was observed between niche overlap and DNA barcodes, indicating that spatial distribution had a major influence on chemical composition in the *Fritillaria* species. Finally, the acquisition of species-specific habitats would contribute to decrease in habitat competition, and future conservation and cultivation of *Fritillaria* species.

## Introduction

Plants regulate balance between oxygen and carbon dioxide in atmosphere, reduce contents of air pollutants (SO_2_, NO_2_ and VOC), produce pharmaceutical and commercial ingredients for both humans and animals, and are therefore an essential component of ecosystems^1^. Recently, many studies reported that environmental factors (temperature, light, water, etc.) play roles in numerous physiological and metabolic processes in plants, thus substantially affecting the distribution and biodiversity of many plants^2^. On the other hand, the accumulation of plant secondary metabolites, which are regulated by the climate change, plays a role in plant-rhizospheric microbe interactions, thereby making significant contributions to the environmental properties^3^. According to the IPCC record, the global average temperature has continuously risen by 0.78 °C compared to that from 1885 to 1990 due to increased energy consumption and greenhouse gas emission^4,5^. Accordingly, warmer temperatures would impact plant growth and yield^6,7^, leading a rise in the extinction rate of species. However, many plants may not be able to make shift to suitable areas with sufficient rate at the current speed of climate change. Hence, detecting the degree of environmental change in the coming future and assessing its impact on plant distribution may be helpful in designing future conservation and cultivation methods^8^.

*Fritillaria* species are regarded as a well-known symbol of thoughts, which were initially introduced in *Shijing*, the most ancient form of poetry in China 3000 years ago. In addition to aesthetic value, bulbus *Fritillaria cirrhosa* (BFC), also called as the Chinese name “Chuan-Bei-mu”, has been used in traditional Chinese medicine to relieve cough and phlegm for more than 2000 years^9^. In terms of China Pharmacopoeia 2020, the bulbs of *F. cirrhosa*, *F. unibracteata*, *F. przewalskii*, *F. delavayi*, *F. wabuensis*, and *F. taipaiensis* are sources of BFC. Among the six species, *F. cirrhosa*, *F. unibracteata*, *F. przewalskii*, *F. delavayi* and *F. wabuensis* are grouped as high-altitude species distributed in the alpine areas of the Himalayan-Hengduan Mountains, while middle-altitude species (*F. taipaiensis*) is distributed mainly in Shanxi and Chongqing provinces. Recently, the imbalance between supply and requirement of BFC became severe partly due to the unsustainable harvesting of *Fritillaria* species. As per the Information System of Chinese Rare and Endangered Plants (ISCREP) (http://www.iplant.cn/rep/protlist), most wild *Fritillaria* species are endangered as their wild resources are rarely found in China. The beneficial ingredients of *Fritillaria* species are mainly composed of steroidal alkaloids^10^, whose biosynthesis mainly originates from the mevalonate (MVA) pathway^11^. The genes involved in the MVA pathway have been shown to be regulated by environmental factors, with those in the distribution area perhaps affecting their chemical composition and potential applications^12^. In addition, altitude serve as an important aspect affecting the biological growth and morphological plasticity of *Fritillaria* species^13^. Meanwhile, the high-altitude *Fritillaria* species may be a commercially important genetic pool as these plants are highly adapted to cold and dry climates^14^. All of these results have confirmed the important and active role of environment in plant physiology, productivity, and other processes. Therefore, it is vital to explore the correlation between the environmental variables and the distribution of *Fritillaria* species, and determine the key factors influencing its distribution.

Two kind of species distribution models (SDMs), such as maximum entropy (MaxEnt) and genetic algorithm for rule-set production (GARP), have been singly used to identify important environmental variables, ecological responses, and distribution areas^15,16^. Recently, ensemble models have been proposed as they may improve the accuracy compared to single models^17^. Regardless of modeling approach, area under the curve (AUC) of the receiver operating characteristic (ROC) curve, true skill statistics (TSS), and Kappa values, are usually used as evaluating criteria for confirmation of final model^5^. In our previous study, we predicted the potential distribution of *F. unibracteata*, *F. cirrhosa* and *F. przewlkii* by Maxent and GARP^5^; however, little is known regarding the other three *Fritillaria* species. In this study, the potential distribution of *F. taipaiensis*, *F. delavayi* and *F. wabuensis* were analyzed, which may provide more scientific support for the cultivation and conservation of *Fritillaria* resources. First, the occurrence records of *F. delavayi*, *F. taipaiensis* and *F. wabuensis* are collected. After evaluating the accuracy of various models, an ensemble model using Biomod2 package of R software is finally selected to assess what degree of environmental variables affecting the potential distribution range of the three *Fritillaria* species^17–19^. Moreover, in order to avoid the habitat competition of various *Fritillaria* species, the overlapping distribution and species-specific habitats of various original species is then tested. Second, the potential change in ecological distribution under four climate scenarios (SSP126, SSP245, SSP370, and SSP585) is predicted, by which sustainable strategy might be developed prior to the future trend happens. Third, it is hypothesized that the environmental distribution shows influence on the genetic information and chemical composition of plants, and therefore the correlation between spatial distribution and DNA sequences or chemical composition was evaluated, respectively.

## Materials and methods

### Occurrence records

The occurrence records of three *Fritillaria* species across their whole ranges in China were collected through the following sources: field surveys since 2017, related literature searching, and websites (Global Biodiversity Information Facility, GBIF, https://www.gbif.org/; Chinese Virtual Herbarium databases, CVH, https://www.cvh.ac.cn/; Plant Science of China, http://www.iplant.cn/frps). In total, we used records (temporal range of records: 1884–2020). When distribution points did not contain the exact geo-coordinates, the Chinese Satellite Map (https://map.bmcx.com/) was used to pick up the latitude and longitude. The repeated records were removed with only one point within each grid cell (20 km × 20 km). ArcGIS (v10.2, ESRI, Redlands, CA, USA) was utilized to analyze the actual distribution, richness, and diversity of three *Fritillaria* species. By using ArcGIS, all the present points of *F. delavayi*, *F. taipaiensis*, and *F. wabuensis*, were recorded and mapped a decimal degree format with 1° × 1° resolution, respectively.

### Environmental parameters

In this study, 19 bioclimatic variables (bio01-bio19) with 2.5 arc-min spatial resolution from the WorldClim database version 2.0^20^, and three topographic variables (slope, altitude, and aspect extracted in ArcMap, v10.2, https://www.arcgis.com/index.html) were initially selected. The 22 environmental variables (Supplementary Table 1) have been broadly used to explore environmental influences on alphine plants in the Himalayan-Hengduan Mountains^21,22^.

Prior to model construction, Spearman correlation analysis among the 22 environmental variables was undergone in order to avoid model overfitting. Specifically, using the aforementioned distribution points of *Fritillaria*, a correlation matrix using SPSS 2.0 was performed so as to extract the associated environmental variables. Then, in line with the methods outlined by Jiang et al., if paired environmental variables showed a coeffiecient of correlation > 0.8, the one with a higher contribution rate was subsequently remained (Supplementary Table 2) and used for further prediction^23^.

In order to analyze the influence of climate change on future species distribution, four shared socioeconomic pathways (SSP126, SSP245, SSP370, and SSP585) released by the CMIP6 and IPCC Assessment Report 6 (AR6) were applied, which represent the pathways of global sustainable, medium sustainable, local sustainable, and routine development, respectively. Based on the World Climate Database^24^, global projection was presented at a spatial resolution of 2.5 arc-min from 2021 to 2100 (20-year intervals). All environmental variables in raster format were aligned with the geographic space using WGS84 datum as the default parameters.

### Evaluation of model and potential distribution of *Fritillaria* species

MaxEnt (v3.3.1, https://biodiversityinformatics.amnh.org/open_source/maxent/), GARP (v1.1.6, http://www.nhm.ku.edu/desktopgarp/) and Biomod2 (v4.3.1, https://cran.r-project.org/bin/windows/base/) models were initially used. Biomod2 is an ensemble SDM method capitalizing on ten singly used modeling techniques: artificial neural networks (ANN), classification tree analysis (CTA), flexible discriminant analysis (FDA), generalized additive models (GAM), generalized boosted models (GBM), generalized linear models (GLM), multivariate adaptive regression splines (MARS), MaxEnt, random forest (RF), and surface range envelope (SRE)^25^. In this study, surface range envelope strategy was used, and as a result, pseudo-absence points were randomly generated with the same number as true-presence records^26^. Then, eight commonly used algorithms were applied: GLM, RF, MaxEnt, ANN, MARS, FDA, CTA and SRE^27^. A model option in Biomod 2, each algorithm was run 15 times, for a total of 120 runs for the eight algorithms. In the data sets, various training/testing sets (85/15, 80/20 and 75/25) were conducted using 500 iterations in order to validate the models. Following initial tests, only modeling techniques with a ROC value > 0.9 were left to construct the final ensemble model. The test samples were selected using the bootstrap method.

The accuracy of the MaxEnt, GARP and Biomod2 models was evaluated by AUC, TSS, and Kappa values. For AUC analysis, the Jackknife method was selected to produce the response curve of the environmental variables. Then, after implementing 10 times repetition for each test, the AUC value was counted as the area enclosed by the ROC curve. AUC < 0.6 indicated failing performance, 0.6 ≤ AUC < 0.7 indicated poor performance, 0.7 ≤ AUC < 0.8 indicated moderate performance, 0.8 ≤ AUC < 0.9 indicated good performance, and 0.9 ≤ AUC < 1 indicated excellent performance^28^. TSS scores ranged from − 1 to 1, where values above 0.75 indicated excellent model performance^29^. Kappa coefficients were measures of correlation between the model predictions and truth. Kappa values ranged from − 1 to 1, where 1 indicated that 100% of prediction agrees with the truth, − 1 indicated that 100% of prediction disagreed with the truth^30^.

The prediction results under the optimal model were then imported into ArcGIS after which the current suitable habitats for three *Fritillaria* species were drawn. During ArcGIS mapping, four grades of suitable habitats were classified: highly (0.75–1), moderately (0.5–0.75), low (0.25–0.5), and not suitable habitat (0–0.25)^31^. Since the suitable habitats with probability ≥ 0.5 indicated that species lived comfortably in these areas^32^, the suitable ranges of the environmental variables were defined with a threshold of ≥ 0.5^33^. The corresponding values of suitable range were acquired from the response curve of the Biomod2 model result.

### Niche overlap of *Fritillaria* species

First, the mean Levins’ B1 (inverse concentration) and B2 (uncertainty) values^34^ were calculated as the niche breadth of each species in geographical and environmental space using ENMTools v1.3^35^. The ranges of Levins’ B1 and B2 values distributed from 0 to 1, where higher values represented wide niche breadth, while lower values represented narrow niche breadth. Then, the niche overlap was evaluated using ENMTools package based on two indexes: Schoener’s D^36^ and Hellinger’s I^35^, whose values generally ranged from 0 (representing a small extent of niche overlap) to 1 (representing a high extent of niche overlap).

Finally, to strengthen the speculation, the overlapping regions including highly and moderately suitable habitats of paired species were visualized in ArcGIS, whose overlapping extent was counted according to the following equation^5^.

$$ Overlapping\;derree = \frac{{A_{overlap} + B_{overlap} }}{{A_{total} + B_{total} }} $$

A_overlap_ and B_overlap_ represent the records of species A and B in the overlapping regions respectively, while A_total_ and B_total_ are the total existing records related to species A and B.

### Clustering analysis of *Fritillaria* species based on chemical metabolites

The cluster analysis based on the chemical composition of six *Fritillaria* species was performed in term of the method reported by An et al.^37^. Specifically, the chemical metabolites of *Fritillaria* species were obtained from the Traditional Chinese Medicine Integrated Database (TCMID, http://119.3.41.228:8000/tcmid/), which is currently the largest database, containing 25,210 secondary metabolites from 8159 herbs^38^. 40 chemical metabolites were extracted from five *Fritillaria* species in TCMID, of which 31 non-repetitive metabolites had SMILES information. Since the chemical metabolites information of *F. taipaiensis* was not contained in TCMID, four alkaloids of *F. taipaiensis* with potential medical values were collected from academic literature and were introduced into this study (Supplementary Table 3). Using the PaDel-descriptor software, the SMILES information for each metabolite was transformed into a PubChem binary fingerprint (Supplementary Table 4). PubChem contains 881 bits and has been widely used in chemo-informatics systems^39^. Next, a metabolites network was constructed in light of chemical structure similarity^40^, in which paired metabolites with Tanimoto coefficient value greater than 0.85 were considered to be highly similar in structure^41^. Third, metabolites with similar structures were clustered to produce metabolite-groups based on IPCA algorithm^42^. Fourth, a binary matrix between the metabolite-groups and plants was built, and accordingly the plants were clustered by Simpson coefficient^43^ and Jaccard coefficient^44^, respectively. Finally, the plant-plant dissimilarity in Jaccard clustering was used for the correlation analysis.

### Phylogenetic analysis

In order to reveal the evolutionary relationship among the *Fritillaria* species, the ITS1, ITS2, ITS1 + ITS2 and complete chloroplast genomes of the six original species of BFC were collected from GenBank, some of which were deposited by our team (Zhang et al.). Phylogenetic trees based on Maximum likelihood were constructed using MEGA 7.0 with 1000 bootstrap repetitions^45^. The evolutionary distance, derived from nucleotide substitution rates based on the Tamura-Nei model^46,47^, was used for the further correlation analysis^48^.

### Statistical methods

A linear trend line representing the correlation between evolutionary distances from DNA markers or plant-plant dissimilarity based on chemical composition and niche overlap was drawn. The *R*^2^ value indicates how well the data fit the line, where 1 signifies a strong correlation, while 0 refers to poor correlation. A Pearson correlation test was applied to check the statistical significance with a *P*-value of ≤ 0.05 to be statically significant^49^.

## Results

### The occurrence records of three *Fritillaria* species

**Figure 1:**
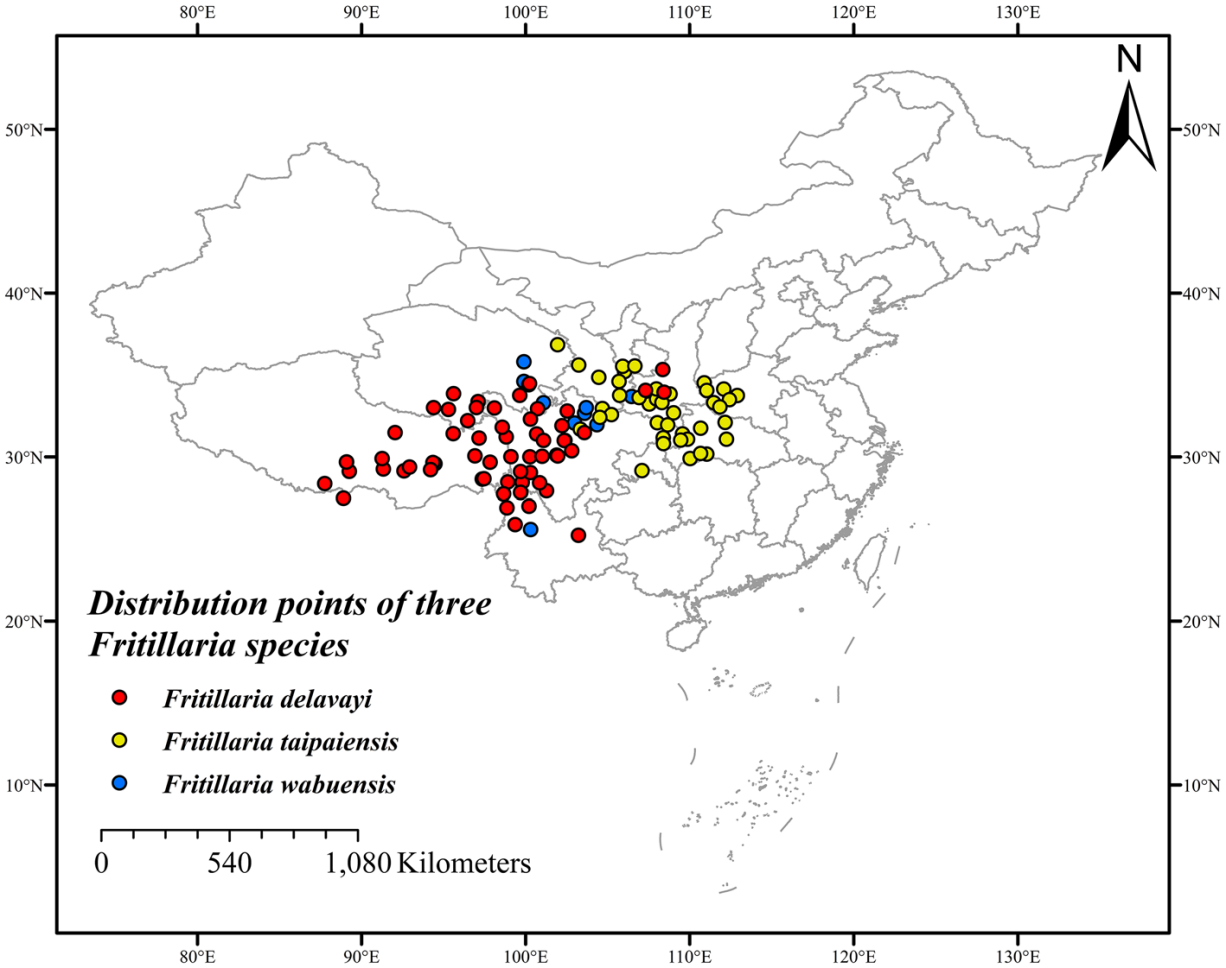
Distribution points of *F. delavayi* (red), *F. taipaiensis* (yellow) and *F. wabuensis* (blue), respectively. MaxEnt v3.3.1: https://biodiversityinformatics.amnh.org/open_source/maxent/, ArcGIS v10.2: https://www.arcgis.com/.

Initially, a total of 147 distribution points were collected on three Fritillaria species by field survey, literature and websites search. Following refinement, 29 distribution points were removed and the remaining 118 individual sample points were recorded, of which 56, 45, and 17 records of *F. delavayi*, *F. taipaiensis*, and *F. wabuensis* were collected, respectively (Supplementary Table 5). In accordance to the sampling point map (Fig. 1), the ecological factor data of each sampling point was extracted. These three *Fritillaria* species were found to be distributed in the middle-latitude region (25.23°N–36.84°N). Meanwhile, the average latitudes of *F. taipaiensis*, and *F. wabuensis* were noted to be above 31°N, while that of *F. delavayi* was below 31°N. The distribution spans of three species in longitude were observed to be narrow, and hence their resource distribution was relatively concentrated.

Three species were distributed with an average altitude of more than 1000 m, in which *F. delavayi* showed the highest average altitude of 3395.29 m. The average aspect of *F. delavayi* and *F. wabuensis* was noted to be above 200, while that of *F. taipaiensis* was below 200. The slopes of three *Fritillaria* species were in the range of 88°–90°.

### Model performance and importance of environmental factors

With the given training/testing sets (85/15, 80/20, and 75/25), compared with the MaxEnt and GARP models, the ensemble model Biomod2 was found to have the best performance according to AUC, KAPPA, and TSS values (Supplementary Table 6). When the training/testing set was 85/15, *F. delavayi* (AUC = 0.999, KAPPA = 0.964, and TSS = 0.970) was shown to possess the best performance, when the training/testing set was 80/20, *F. taipaiensis* (AUC = 0.997, KAPPA = 0.956, and TSS = 0.963) and *F. wabuensis* (AUC = 0.999, KAPPA = 0.962, and TSS = 0.980) had the best performances. Therefore, Biomod2 was confirmed to be the optimum model for three *Fritillaria* species, in which *F. taipaiensis* and *F. wabuensis* utilized 80/20 set, while *F. delavayi* utilized 85/15 set in the following analysis.

**Figure 2:**
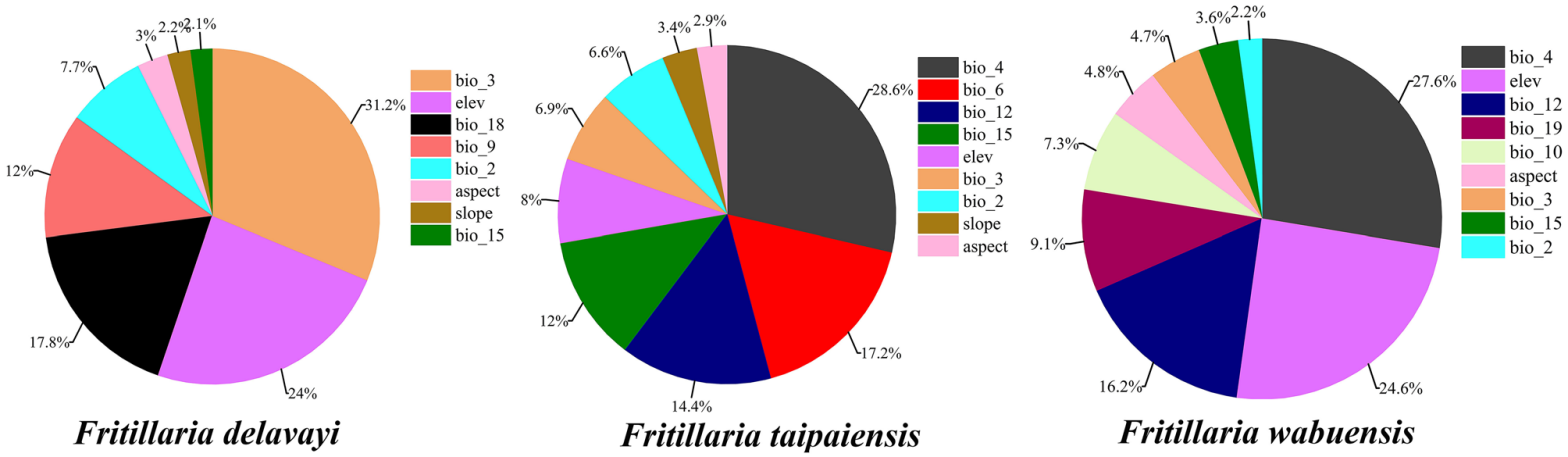
Importance of various environmental variables on three *Fritillaria* species.

The internal Jackknife test determined the important environmental variables (Fig. 2). In case of independent application, Bio3 (31.2%), Elev (24%), Bio18 (17.8%), and Bio9 (12%) were highlighting parameters in the distribution model for *F. delavayi*, accounting for 85% of the variables. Bio4 (28.6%), Bio6 (17.2%), Bio12 (14.4%), Bio15 (12%), Elev (8%), and Bio3 (6.9%) contributed the most to the distribution model of *F. taipaiensis*, accounting for 87.1% of the variables. Moreover, the environmental variables with the highest influence on *F. wabuensis* were Bio4 (27.6%), Elev (24.6%), Bio12 (16.2%), Bio19 (9.1%) and Bio10 (7.3%), accounting for 84.8% of the variables.

**Figure 3:**
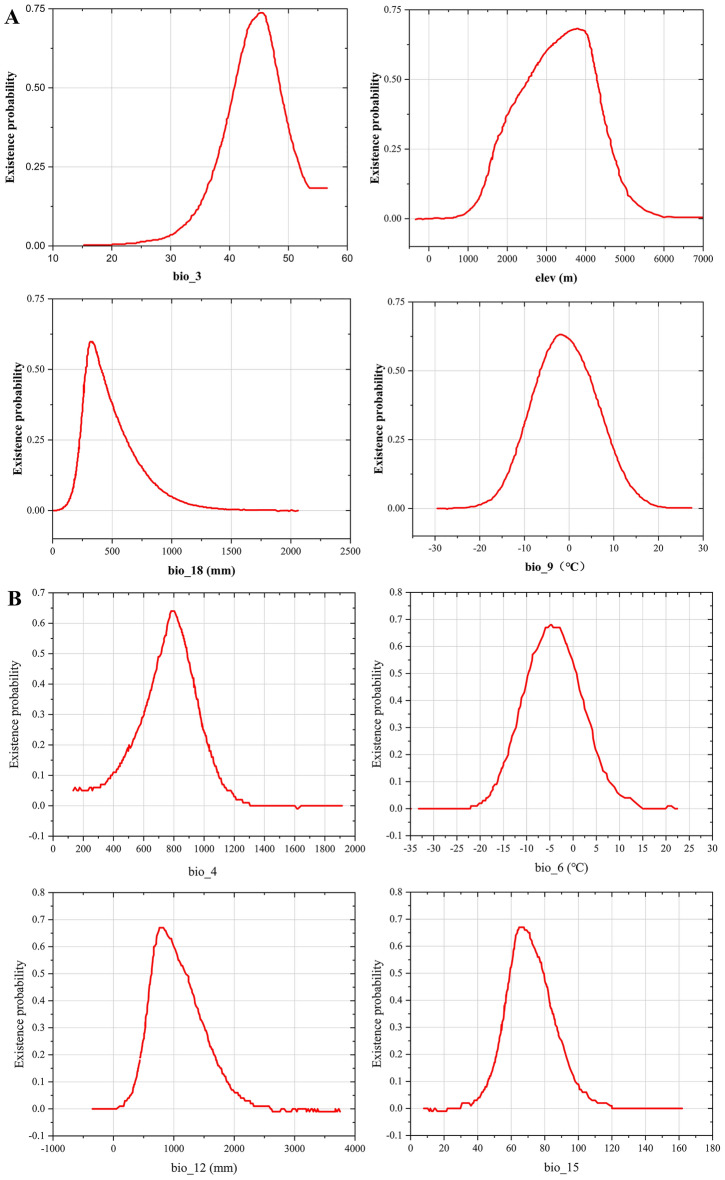

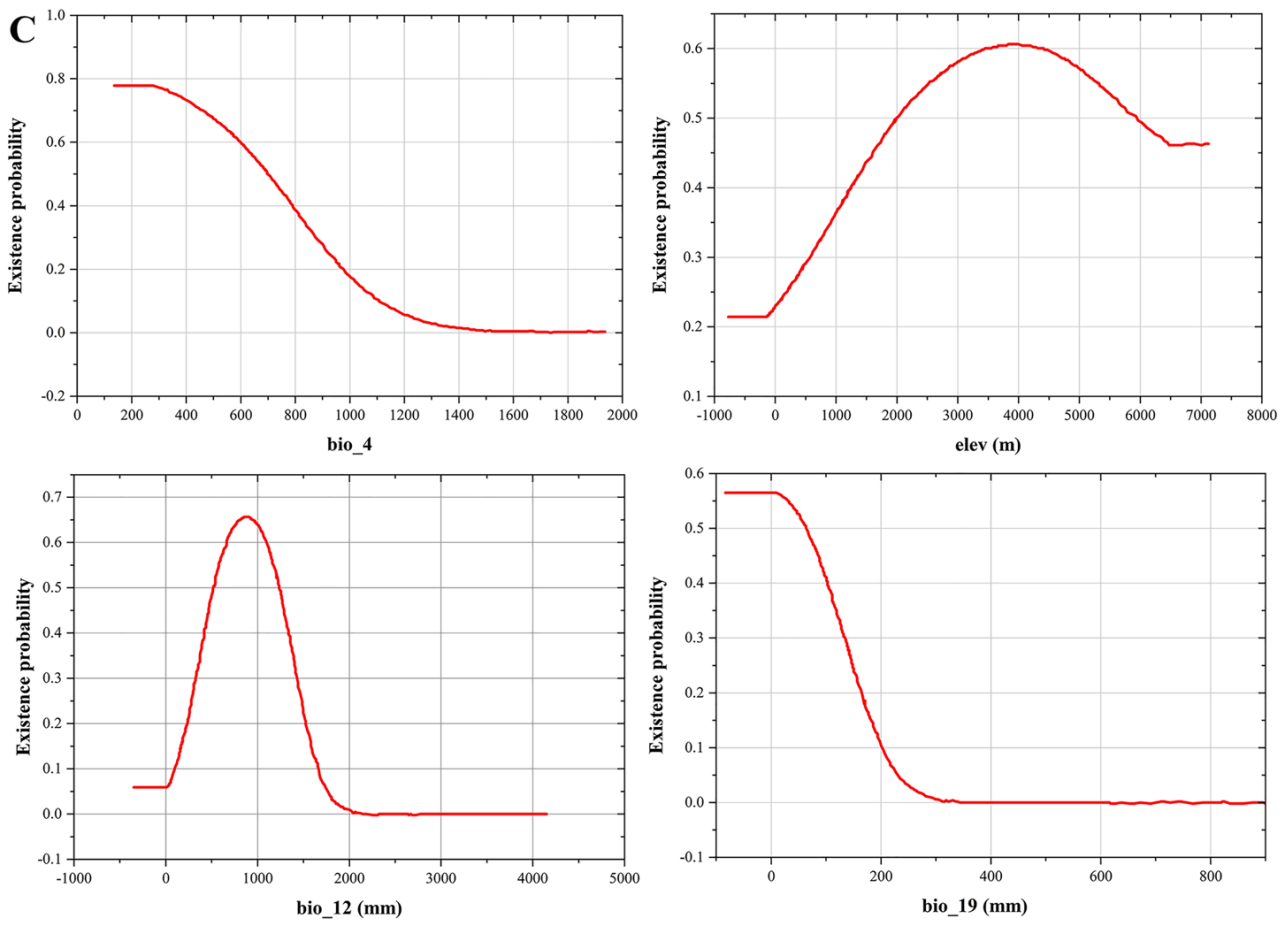
Response curves for dominant environmental variables in the species distribution model for *F. delavayi* (**A**), *F. taipaiensis* (**B**), and *F. wabuensis* (**C**), respectively.

The response curve demonstrated the quantitative relationship between the logistic probability of the presence and suitable ranges of environmental variables (Fig. 3). In terms of the survival areas with moderate and high suitability, the threshold (≥ 0.5) was chosen for the main bioclimatic parameters^50^, since plants live comfortably in such conditions. The existence probability of three *Fritillaria* species was shown to initially increase, after which it decreased with a rise in environmental factors. Specifically, the suitable ranges of Bio3, Bio9, Bio18, and Elev (Table 1) for *F. delavayi* were 40.89–48.59, − 6.49–3.77, 279.07–404.65 mm, and 2554.27–4284.06 m, respectively. Optimal isothermality (Bio3) reflects the regional temperature change and is calculated by the ratio between Bio2 and Bio7. When Bio3 reached 45.47, *F. delavayi* attained the highest existence probability of 0.74. When Bio9 reached the maximum at − 2.16, the existence for *F.delavayi* met the highest probability of 0.63. *F. delavayi* achieved the highest probability of existence of 0.598 as Bio18 reached 334.88 mm. Finally, the top probability of existence (0.68) was achieved once Elev reached 3782.91 m (Fig. 3A), by which confirmed that *F. delavayi* is high-altitude species.

**Table 1:** Suitable range and optimum environmental variables of three *Fritillaria* species.

Species	Environmental variables	Suitable range	Optimum	Maximum probability of existence
*F. delavayi*	Bio3	40.89–48.59	45.47	0.74
	Bio9	− 6.49–3.77	− 2.16	0.63
	Bio18 (mm)	279.07–404.65	334.88	0.598
	Elev (m)	2554.27–4284.06	3782.91	0.68
*F. taipaiensis*	Bio4	715.51–888.61	785.18	0.64
	Bio6 (°C)	− 9.46–0.78	− 4.87	0.68
	Bio12 (mm)	633.38–1196.7	822.12	0.67
	Bio15	59.52–79.53	66.97	0.67
*F. wabuensis*	Bio4	135.98–697.07	279.08	0.78
	Elev (m)	1996.54–5930.37	3828.33	0.61
	Bio19 (mm)	− 83.43–63,56	3.51	0.56
	Bio12 (mm)	521.88–1234.38	850.0	0.66

In *F. taipaiensis*, the suitable range of Bio4, Bio6, Bio12 and Bio15 ranged from 715.51 to 888.61, from − 9.46 to 0.78 °C, from 633.38 to 1196.7 mm, and from 59.52 to 79.53, respectively (Fig. 3B). Moreover, the optimum values of Bio4, Elev, Bio19 and Bio12 were 279.08, 3828.33 m, 3.51 mm and 850.0 mm for *F. wabuensis,* respectively (Fig. 3C), in which the relative high altitude also demonstrated that *F. wabuensis* is high-altitude plant. In respect to *F. taipaiensis*, *F. wabuensis* showed the relative smaller temperature seasonality (Bio4), suggesting that the suitable presence of *F. wabuensis* was associated with lower variability in temperature. Meanwhile, *F. taipaiensis* and *F. wabuensis* shared similar value of annual precipitation (Bio 12), suggesting that both species shared similar water requirement. The suitable ranges and optimum values of environmental variables were listed in Table 1.

### Current habitats of three *Fritillaria* species

**Figure 4:**
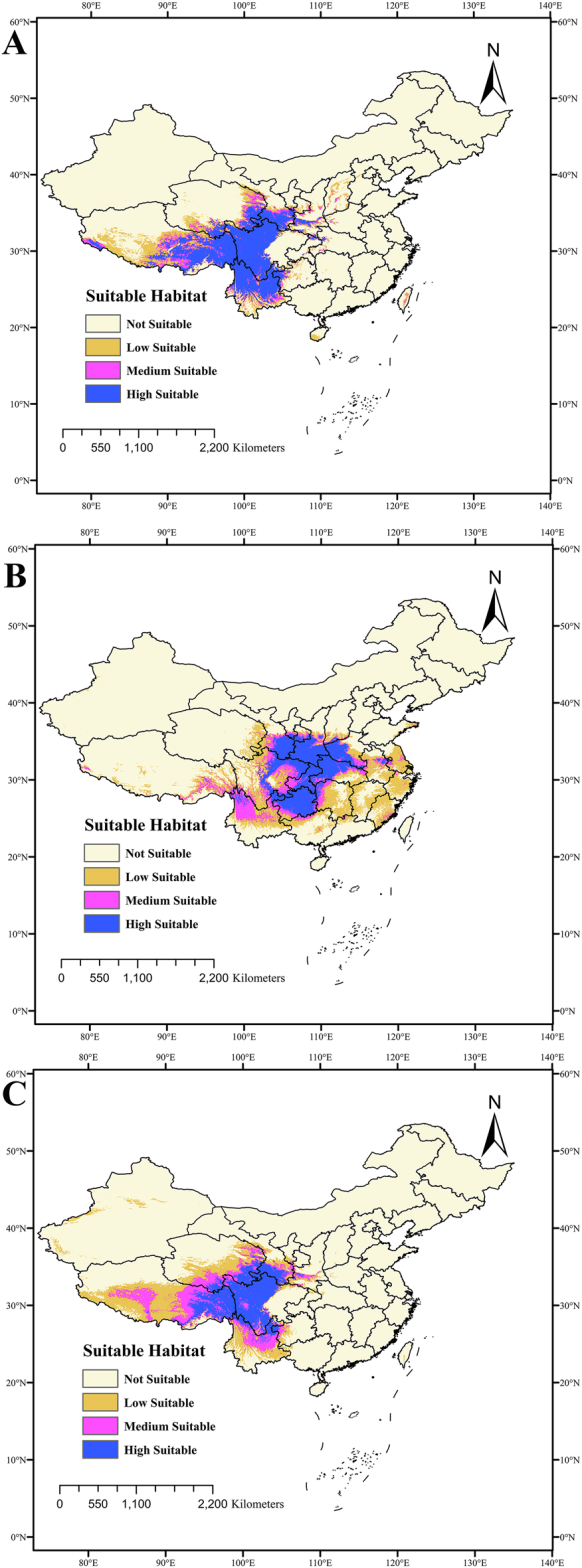
Distribution map of suitable areas for *F. delavayi*, *F. taipaiensis* and *F. wabuensis*, respectively. MaxEnt v3.3.1: https://biodiversityinformatics.amnh.org/open_source/maxent/, ArcGIS v10.2: https://www.arcgis.com/.

The predicting current habitats for *F. delavayi* demonstrated that highly suitable habitats were in Qinghai, Sichuan, Tibet and Yunnan provinces, accounting for 9.48% of China's total area. Moreover, the moderately suitable habitats were found to be distributed in the Qinghai, Tibet, and Yunnan provinces. The total suitable habitats for *F. delavayi* reached 1.70 × 10^6^ km^2^, accounting for 17.72% of China's total area (Fig. 4A). For *F. taipaiensis*, the highly suitable habitat was shown to be distributed in the Gansu, Guizhou, Henan, Hubei, Shanxi, and Sichuan provinces, accounting for 7.03% of China's total area. Meanwhile, moderately suitable habitats included the Anhui, Fujian, Guizhou, Guangxi, Hubei, Jiangsu, Sichuan, Tibet, and Yunnan provinces. The total suitable habitat for *F. taipaiensis* comprised 2.21 × 10^6^ km^2^, accounting for 23.06% of China's total area (Fig. 4B). As for *F. wabuensis*, the highly suitable habitat was shown to be in Gansu, Guizhou, Qinghai, Sichuan, Tibet, and Yunnan, while the moderately suitable habitat was almost in Chongqing, Gansu, Guizhou, Hubei, Shanxi, and Tibet provinces. The current suitable habitat for *F. wabuensis* was 1.98 × 10^6^ km^2^, accounting for 20.60% of China's total area (Fig. 4C). Compared with other two *Fritillaria* species, *F. delavayi* showed the smallest suitable habitat, which was consistent with its endangered state.

### Future changes in suitable habitat area

**Figure 5:**
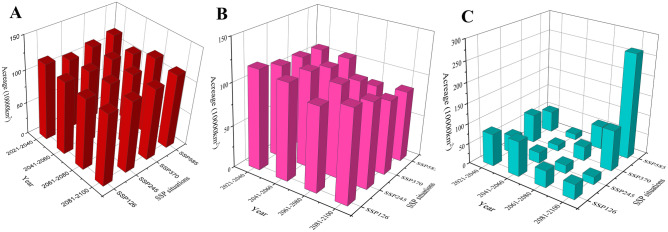
The suitable areas of *F. delavayi* (**A**), *F. taipaiensisi* (**B**), and *F. wabuensis* (**C**), respectively, in China under future climate conditions.

As listed in Supplementary Table 7, in *F. delavayi* (Fig. 5A), the maximum area of highly and moderately suitable habitats reached the maximum (12.14%, 11.77%, 11.90% and 12.49%) under SSP126, SSP245, SSP370 and SSP585 scenarios, respectively. For SSP585 scenario, the maximum in highly and moderately suitable habitat appeared at 2061–2080 period (Supplementary Fig. 2A). Accordingly, SSP585 was confirmed to be the optimum scenario for *F. delavayi*, as its highly and moderately suitable habitat reached the maximum during 2021–2100 at 1199,653.339 km^2^.

In *F. taipaiensisi* (Fig. 5B), the maximum area of the highly and moderately suitable habitat occurred with 12.24%, 11.79%, 11.27% and 11.08% under SSP126, SSP245, SSP370 and SSP585 scenarios, respectively. For SSP126 scenario, the maximum took place during 2021–2040 (Supplementary Fig. 2B). Overall, SSP126 was found to be the optimum scenario for *F. taipaiensis* with the maximum area (1174,666.794 km^2^) in the highly and moderately suitable habitats during 2021–2100.

In *F. wabuensis* (Fig. 5C), the areas of highly, moderately and total suitable habitats increased in a wave-like mode under the four scenarios, respectively. Under the SSP126, SSP245, SSP370 and SSP585 scenarios, the maximum area of highly and moderately suitable habitats was shown to be 9.05%, 4.54%, 10.57% and 27.42%, respectively. The highly and moderately suitable habitats reached the maximum during 2081–2100 under the SSP585 scenario (Supplementary Fig. 2C). SSP585 was shown to be the optimum scenario for *F. wabuensis* since the highly and moderately suitable habitats reached their maximum areas during 2021–2100 at 2632,258.065 km^2^, respectively.

### Ecological niche overlapping of three *Fritillaria* species

As indicated by the highest B1 (0.295) and B2 (0.927), *F. taipaiensis* exhibited the broadest niche, while *F. wabuensis* showed a narrower niche (B1 and B2: 0.260 and 0.916, respectively) and *F. delavayi* had the narrowest niche (B1 and B2: 0.228 and 0.914, respectively). *F. wabuensis* and *F. delavayi* shared the more similarity due to the highest niche overlap (I: 0.938, D: 0.789), while *F. taipaiensis* showed lower similarity with *F. delavayi* (I: 0.756, D: 0.463) and *F. wabuensis* (I: 0.700, D: 0.432).

**Figure 6:**
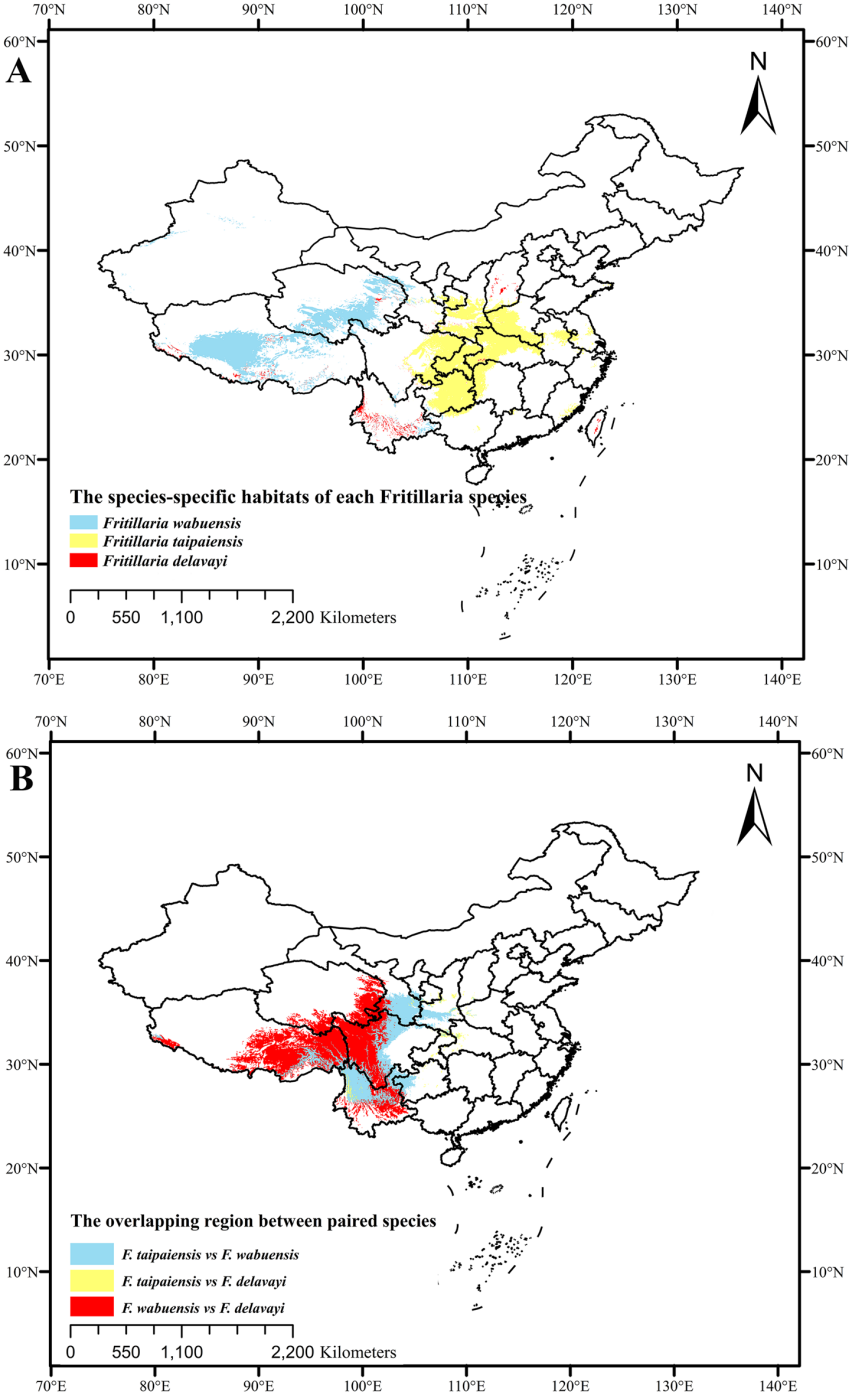
Distribution patterns of three *Fritillaria* species. (A) Species-specific highly and moderately suitable habitats of *Fritillaria delavayi, Fritillaria taipaiensis,* and *Fritillaria wabuensis*. (B) Overlapping region of highly and moderately suitable habitats between paired species. MaxEnt v3.3.1: https://biodiversityinformatics.amnh.org/open_source/maxent/, ArcGIS v10.2: https://www.arcgis.com/.

Then, the overlapping regions for three *Fritillaria* species were counted and drawn in Fig. 6, and hence the species-specific habitat of *F. delavayi* was found to be distributed in the central region of Yunnan province, while that of *F. wabuensis* was mainly distributed in Qinghai and Tibet provinces. Additionally, the species-specific habitat of *F. taipaiensis* was mainly concentrated in Chongqing, Guizhou, Henan, Hubei, Shanxi and Sichuan provinces as shown in Fig. 6A. The overlapping degree between *F. wabuensis* and *F. delavayi* was found to be 58.96%, while that of *F. wabuensis* and *F. taipaiensis* was 15.90% and that of *F. taipaiensis* and *F. delavayi* was 9.98% (Fig. 6B). The results were noted to be consistent with the results reported by ENMTools, in which the highest niche overlap was observed between *F. wabuensis* and *F. delavayi.*

### Correlation analyses on DNA Phylogenies, clustering tree based on chemical metabolites with niche overlap of three *Fritillaria* species

**Figure 7:**
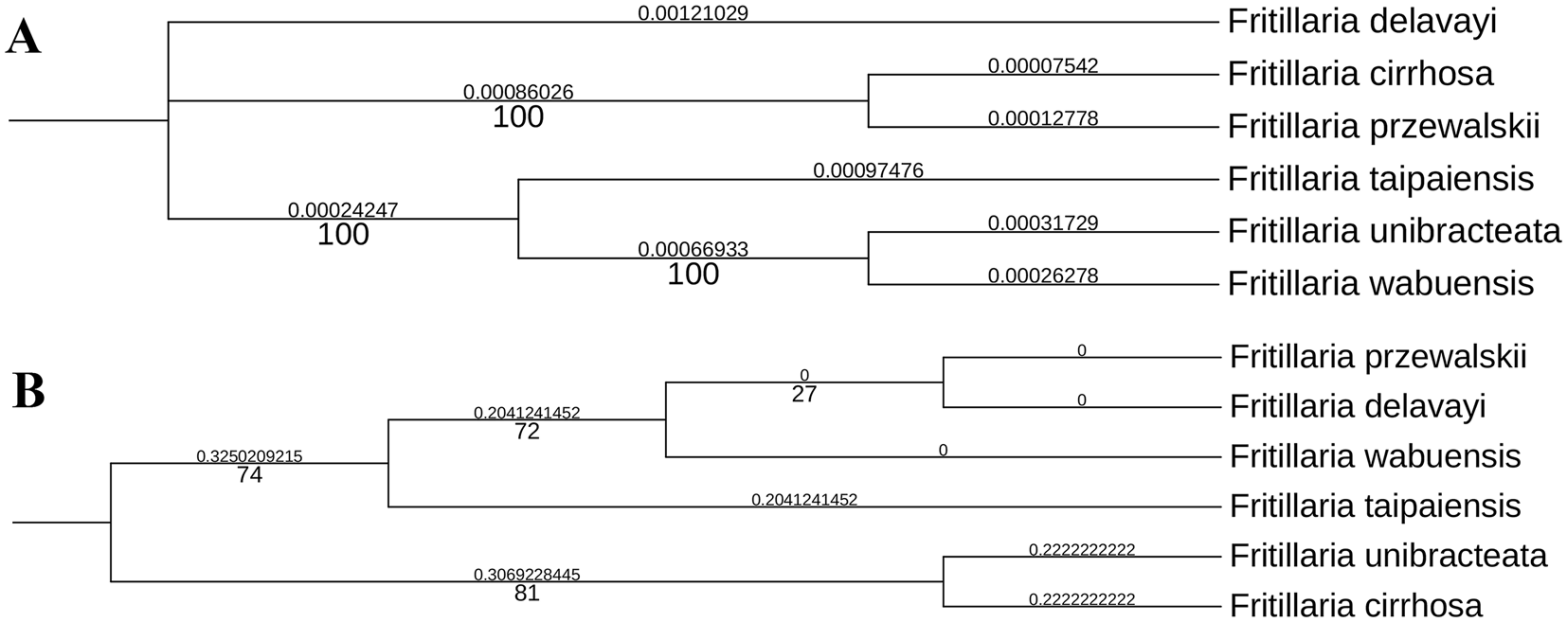
Phylogenetic trees based on CP genomes and the Jaccard clustering tree based on dissimilarity of chemical composition. The pairwise distance (above the branches) and bootstrap value (under the branches) were listed. (**A**) Phylogenetic tree based on CP genomes; (**B**) Jaccard clustering tree based on dissimilarity of chemical composition.

Compared with ML trees based on ITS1, ITS2, and ITS1 + ITS2 that were weakly supported (Supplementary Fig. 1), that based on the whole CP genome exhibited higher support since three branches got bootstrap values of 100 BP (Fig. 7). Therefore, the CP genome was used to to evaluate correlation, though no correlation between evolutionary distance and niche overlap was observed (*P* > 0.05). This may be because genetic evolution is slower than changing speed in species distribution, or because fritillary distribution is influenced by artificial planting preferences.

On the other hand, two clustering trees based on the chemical composition were separately constructed. Compared with the Simpson tree, the Jaccard tree received higher support as three branches got bootstrap values of more than 70 BP, while branches in Simpson tree had bootstrap value of lower than 70 BP. In the Jaccard tree, six original species of Chuan-Bei-mu were grouped into two clades, in which clade 1 was composed of *F. unibracteata* and *F. cirrhosa,* and clade 2 was composed of other four *Fritillaria* species. In clade 2, *F. taipaiensis* was the sister to the other three species and showed a higher dissimilarity with *F. delavayi* and *F. wabuensis*. Using Jaccard result, the correlation between nichel overlap and chemical composition was evaluated, and hence a strong correlation was observed (R^2^ = 0.994, *P* = 0.050) (Table 2). The corresponding findings suggested that the chemical composition of various species was influenced by different spatial distributions.

**Table 2:** The correlation between DNA phylogeny, clustering tree based on chemical composition and spatial distribution.

Type	Niche overlap	R^2^	*P*-value	Niche overlap		
				*F.delavayi* vs.*F.taipaiensis*	*F.delavayi* vs.*F.wabuensis*	*F.taipaiensis* vs.*F.wabuensis*
CP	D	0.254	0.664	0.463	0.790	0.432
CP	I	0.392	0.569	0.757	0.938	0.700
CC	D	0.994	0.050	0.463	0.790	0.432
CC	I	0.949	0.145	0.757	0.938	0.700

CP represents DNA phylogeny based on chloroplast genomes, while CC represents clustering tree based on chemical composition.

## Discussion

Although BFC has been used for healthy treatment for more than two thousand years, the insufficient amount of research conducted on BFC has hindered its sustainable conservation and application. In this study, the suitable habitats of three *Fritillaria* species were analyzed in detail under current and future climate scenarios. Accordingly, the spatial distribution of the *Fritillaria* species was found to strongly show influence on its chemical composition. The corresponding results could serve as an important step in making a sustainable strategy for future conservation.

Prior to the final model’s construction, the effectiveness of various models was significant in obtaining accurate prediction results. With respect to a single model, including Maxent and GARP, the ensemble model, Biomod2 was found to have the best performance due to its highest AUC, KAP and TSS values for each *Fritillaria* species, respectively. Previous analyses^51–53^ have shown that the prediction relied on a single forecast model, which severely compromised its usefulness. Even if the same dataset were used, the different single model achieved various predicting results. A potentially useful pathway may be the development of hybrid approaches, such as Biomod2, which combines a suite of algorithms using the same set of initial data and parameterization^17,52^. Biomod2 has been successfully applied in the distribution prediction of cocoa^54^, *Fagus sylvatica*^55^, lemurs^56^, and *Orientocoluber spinalis*^18^.

The distribution of three *Fritillaria* species represented obvious species-divergent distribution areas since *F. delavayi*, *F. taipaiensis* and *F. wabuensis* were arranged from west to east. *F. delavayi* was found to have a wide distribution with high altitude of 3395.29 m, including central and east Tibet, west Sichuan, north Yunnan, and south Qinghai provinces. The distribution area of *F. delavayi* was also in accord with a study that found that this species is confined to high-altitude medicinal plants^21^. The highly and moderately suitable habitat areas was shown to encompass 1.21 × 10^6^ km^2^ for *F. delavayi* under current climate conditions. Intriguingly, certain moderately suitable habitats in Hubei and Shanxi provinces were also proposed to be introduced of *F. delavayi* in the future. Furthermore, *F. wabuensis* was observed to have the smallest number of distribution records mainly in northwest Sichuan and southwest Qinghai, with sporadic distribution in the Tibet and Yunnan provinces, and exhibited a similar geographic distribution as that of *F. delavayi*. The highly and moderately suitable habitats of *F. wabuensis* encompassed 1.19 × 10^6^ km^2^, ranging mainly from Gansu, Guizhou, Qinghai, Sichuan, Tibet, and Yunnan provinces. The sporadic areas of the Guizhou and Yunnan provinces as moderately suitable habitats may also introduce this species in the future. In comparison with those of *F. delavayi* and *F. wabuensis*, *F. taipaiensis* moved east and was more intense in Chongqing, central and south Shanxi, northwest Hubei, southeast Gansu, and west Henan provinces, and exhibited the smallest highly and moderately suitable areas (1.16 × 10^6^ km^2^) under current climate conditions.

Among various environmental factors, temperature plays the most important role in controlling the distribution of three *Fritillaria* species, accounting for 50.9%, 59.3% and 41.8% overall contribution towards *F. delavayi*, *F. taipaiensis* and *F. wabuensis*, respectively. Specifically, Bio3 in *F. delavayi* (31.2%), Bio4 in *F. taipaiensis* (28.6%) and *F. wabuensis* (27.6%) provided the biggest contribution to the distribution. Moreover, Bio6 provided the second biggest contribution to the distribution of *F. taipaiensis* (17.2%). Temperature was reported to affect bulb dormancy, ovule fertilization, photosynthesis, bulb yielding and total alkaloid contents of the *Fritillaria* species^10,42,57,58^. Supporting that optimum temperature seasonality ranged from 785.18 and 279.08 for *F. taipaiensis* and *F. wabuensis*, respectively, both species showed high levels of probability of presence. This fact confirmed that climatic characteristics shaped population and regeneration of species^59^. Additionally, the relatively wide Bio4 implied that *F. wabuensis* might be adaptive to various areas with broad temperature fluctuation, which aligned with Qin et al.^14^, who indicated that alpine plants showed tolerance to environmental stresses. Meanwhile, low temperature seasonality was found to be consistent since most of the distribution areas of both *Fritillaria* species are located in the Hengduan Mountains and its neighboring regions, most of where have mild to warm winters and temperate summers^60^. In addition to thermal seasonality, isothermality also played a role in the distribution of *F. delavayi*, having an optimum value of 45.47. The large isothermality ensured that *F. delavayi* can not only improve photosynthesis during the daytime due to the relatively high temperature, but reduce energy consumption of respiration due to the relatively low night temperature, which is beneficial for metabolite accumulation and growth^42^. A similar result was recently reported by Wang et al.^61^ who proved that isothermality made the highest contribution to *Fritillaria cirrhosa*. The optimum Bio6 of − 4.87 °C for *F. taipaiensis* suggested a physiological limit to its distribution. In the seeds of *F. taipaiensis*, cold stratification (0–10 °C) was found to break dormancy, while a temperature > 15 °C was unfavorable for decreasing degradation of the substance with germination-inhibiting activities and inhibited germination. Similar results were also obtained by Luo et al.^62^ and Zhang et al.^63^ who observed that cryogenic treatment was necessary to completely break the dormancy of *F. taipaiensis* and *F. yuzhongensis* seeds. On the contrary, relatively high temperature is suitable for seedling growth in *F. taipaiensis*^62^. In addition, suitable temperature plays role in sprout development, morphological maintaining and reproductive process of *Fritillaria* species^64^.

In respect to temperature, precipitation played a relatively minor role in distribution, accounting for 19.9%, 26.4% and 28.9% overall contribution to *F. delavayi*, *F. taipaiensis* and *F. wabuensis*, respectively. Similar result was also reported by Jiang et al.^5^ who observed less contribution of precipitation to the distribution of *F. unibracteata*, *F. cirrhosa* and *F. przewalskii*, indicating the similar water requirement of six original plants of Chuan-Bei-mu. Precipitation implements a considerable influence on the above- and underground organs of plants. Insufficient water has previously been reported to induce a reduction in biomass of plants^65^, longer germination period^66^, lower leaf mass^65^ and lower root density^67^, whereas sufficient water would increase root length and leaf area^45^ and promote germination^68^. In the case of low annual precipitation (< 633.38) for *F. taipaiensis*, low annual precipitation (< 521.88 mm) for *F. wabuensis*, and low Bio18 (< 279.07 mm) for *F. delavayi*, the presenting probability of *Fritillaria* species greatly reduced, indicating that precipitation was an important constraint to both species. Specifically, the annual growth period of *Fritillaria* species was divided into two stages that exhibited different precipitation demands. Generally, precipitation in the first stage (October to next April) was noted to be relatively low and accounted for 20% of the annual demand. Excessive water supplement was found to be unfavorable for cold stratification, which would aggravate *Fritillaria* rust and root rot. Furthermore, freezing rain could cause cellular oxidative damage, dehydration and destabilization to plants^69,70^. These evidences were consistent with our result that *F. wabuensis* got a low value of optimal Bio19 (3.51). Accordingly, low precipitation in the autumn was shown to be beneficial in preventing frost damage to the *Fritillaria* species. Next, abundant precipitation (80% of annual precipitation) in the second stage (May to September) implied that it was necessary to provide sufficient water during the bulb filling period. The balanced water supplement in this stage could improve metabolism turnover, organ formation as well as nutrient accumulation in plants. In general, the relatively low contribution of precipitation was supported by the fact that most of the distribution area, including the center area, of three *Fritillaria* species received the influence of monsoon from the Indian and Pacific oceans and has sufficient precipitation^5^.

Besides, altitude was the third important environmental factor controlling the distribution of three *Fritillaria* species, accounting for 24%, 8% and 24.6% overall contribution to *F. delavayi*, *F. taipaiensis* and *F. wabuensis*, respectively. The accumulation of metabolites and nutrients in underground and subaerial organs are influenced by altitude usually interacting with the availability of nutrients, water content^71^, temperature, ultraviolet intensity, CO_2_ concentration and UV-B^33^. For instance, the reproductive success of *F. delavayi* was critically affected by the temperature and ultraviolet intensity, and as a result, *F. delavayi* was distributed in a narrow altitude area^57^. Ma et al. reported that altitude influenced the growth of leaf and bulb traits, and the biomass of *F. przewalskii* bulb increased significantly with increasing altitude. In this study, the altitude of *F. delavayi* was found to range from 2554.27 to 4284.06 m, which was consistent with^21^, in which *F. delavayi* was confined to be an alpine plant. The similarly high altitude of *F. delavayi* (3782.91 m) and *F. wabuensis* (3828.33 m) ensured that both species could be adaptive to similar alpine environments. As a result, altitude was considered to be one significant factor affecting morphological change and distribution of the *Fritillaria* species^13,50^.

With increasing concentrations of greenhouse gas emissions, the suitable habitat range of *F. taipaiensis* and *F. wabuensis* increased despite varying sensitiveness. Under the low emission (SSP126) scenario, the highly and moderately suitable area of *F. taipaiensis* reached the maximum of 101.1% with respect to those in the current situation (Supplementary Fig. 2B), and *F. wabuensis* reached the maximum of 221.4% under the high emission (SSP580) scenario (Supplementary Fig. 2C). The corresponding findings confirmed that temperature positively influenced plants by accelerating growth rate, increasing size, implicating host resistance to pests, and improving soil nutrients^72,73^. On the other hand, the highly and moderately suitable area of *F. delavayi* was reduced by 1% under the high emission (SSP585) scenario, with respect to those in the current situation (Supplementary Fig. 2A), implying that continuous rise in temperature may confer a negative influence on plants, which may occur by hindering the regeneration of seeds^74^. Accordingly, temperature was determined to be the most significant factor shaping the distribution of the three *Fritillaria* species, which was consistent with Bio4, Bio3 and Bio6 having the most contribution to various species.

Ecological niche was another factor showing influence on the distribution of species. As determined by high Schoener’s D and Hellinger’s I, the high niche overlap among three *Fritillaria* species demonstrated that these species shared similar temperature, precipitation and elevation. Moreover, overlapping areas among three *Fritillaria* species were observed in Fig. 6, and approximately 58.96% of the distributions of *F. delavayi* were located in the overlapping area, confirming the high ecological niche among the three species. Taking that plants with close relationship usually share similar adaptive mechanism to living environments during evolution into consideration^75^, genetic relationship might correlate with ecological niche overlap. However, the spatial distribution was found to be poorly related to phylogeny with weak significance (Table 2). In this regard, diversification and speciation process were proposed to be caused by multiple driving factors, i.e., spatial distribution, reproductive isolation, sexual reproduction and geographical barrier^76–78^. Hopefully, if more factors that are involved with species evolution are applied into the correlation analysis, the statistical result would be more reliable. Additionally, the accumulation of chemical metabolites was influenced by environmental factors; hence, plants sharing common environmental factors may contain a similar chemical composition. In this study, the clustering result that was derived according to the chemical composition from various *Fritillaria* species were found to be correlated with their ecological niche overlap (*P* ≤ 0.050), suggesting that spatial distribution was an important factor in controlling chemical composition. However, unbalanced distribution of metabolites in various plants was observed, i.e., *F. wabuensis* was associated with only one metabolite. Meanwhile, *F. delavayi* was associated with seven metabolites with potential medical values. One reasons for this was that wild medicinal plants (such as *F. delavayi*) contain more compounds compared to cultivated plants (such as *F. wabuensis*), which have been selected artificially. Besides, more systematical studies were possibly carried out on the chemical information of important medicinal plants. Therefore, the relationship between spatial distribution and chemical composition might hopefully be more accurate in light of sufficient chemical metabolite samples.

However, although the suitable planting areas represented regions showing similar environmental conditions as those of current distribution region, economic or other environmental factor variations were not considered in this study. Specifically, the salt in soil was noted to be correlated with soil microbial communities that interacted with the *Fritillaria* species, thereby, influencing its healthy growth^79^. Moreover, excessive consumption of certain medicinal species could lead to extinction in spite of the current and (or) future available suitable habitats. Land use alteration has also resulted in decreasing availability of suitable habitats due to human use for economic purposes^33^. Declined forest cover and increased fragmentation in highly forested reserves may result in the disappearance of large amounts of currently suitable habitats because of ecological distribution^80^ Fortunately, most suitable areas of the three *Fritillaria* species were located in the newly established Giant Panda National Park and Sanjiangyuan National Park, China, which would be able to provide funds and actively manage the conservation of wild species, including *Fritillaria*, in both areas.

## Conclusion

*F. delavayi*, *F. taipaiensis* and *F. wabuensis*, ranked as traditional Chinese Medicine, are widely used and have potentially major market value. However, their resources have rapidly decreased in light of excessive harvesting and insufficient conservation management, and hence information pertaining to suitable areas of the three *Fritillaria* species are urgently required. In this study, ecologically suitable habitats of three *Fritillaria* species were successfully predicted, which were mainly distributed in south-west Asia. Temperature was identified as the most important factor shaping the distribution of the three *Fritillaria* species, however, continuous rise in temperature due to greenhouse gas emissions would cause negative effect on *Fritillaria* species. The ecological niche overlap and species-specific distribution areas among the three *Fritillaria* species may serve as a useful reference in improving future plant introduction and cultivation.

### Supplementary Information

Supplementary Figure 1.

Supplementary Figure 2.

Supplementary Table 1.

Supplementary Table 2.

Supplementary Table 3.

Supplementary Table 4.

Supplementary Table 5.

Supplementary Table 6.

Supplementary Table 7.

Supplementary Information 1.

## Data Availability

The data and materials used and/or analyzed during the current study are available from the corresponding author on reasonable request.
