# Supplementary material for: Biomod2 modeling for predicting the potential ecological distribution of three Fritillaria species under climate change
Source: Sci Rep. 2023 Nov 1;13:18801. doi: 10.1038/s41598-023-45887-6 (PMC10620159; doi:10.1038/s41598-023-45887-6)
Supplement: Supplementary file 1 — Supplementary Figure 1. [file 41598_2023_45887_MOESM1_ESM.pdf]

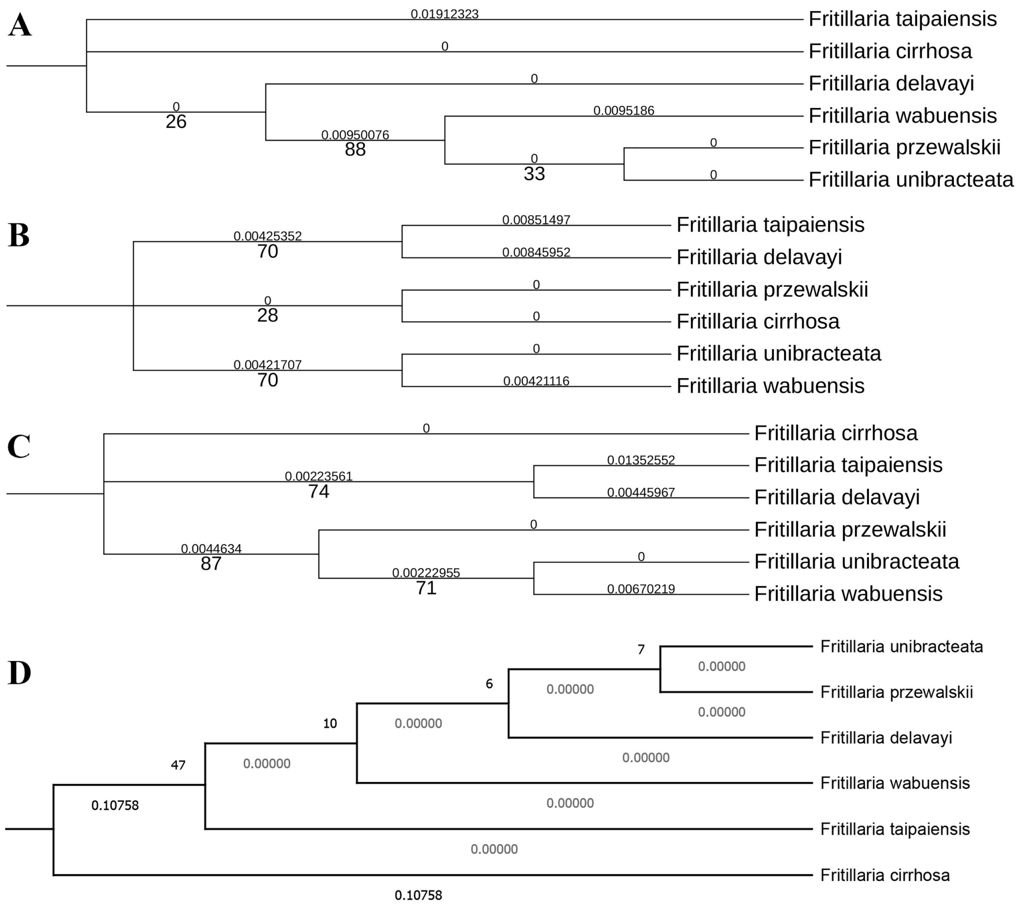

Supplementary Fig 1. Phylogenetic trees based on ITS1, ITS2 and ITS1+ITS2, and Simpson clustering tree based on dissimilarity of chemical composition. The pairwise distance (above the branches) and bootstrap value (under the branches) were listed. (A) Phylogenetic tree based on ITS1 sequences; (B) phylogenetic tree based on ITS2 sequences; (C) phylogenetic tree based on ITS1+ITS2 sequences; (D) Simpson clustering tree based on dissimilarity of chemical composition.
