# Supplementary material for: Biomod2 modeling for predicting the potential ecological distribution of three Fritillaria species under climate change
Source: Sci Rep. 2023 Nov 1;13:18801. doi: 10.1038/s41598-023-45887-6 (PMC10620159; doi:10.1038/s41598-023-45887-6)
Supplement: Supplementary file 2 — Supplementary Figure 2. [file 41598_2023_45887_MOESM2_ESM.pdf]

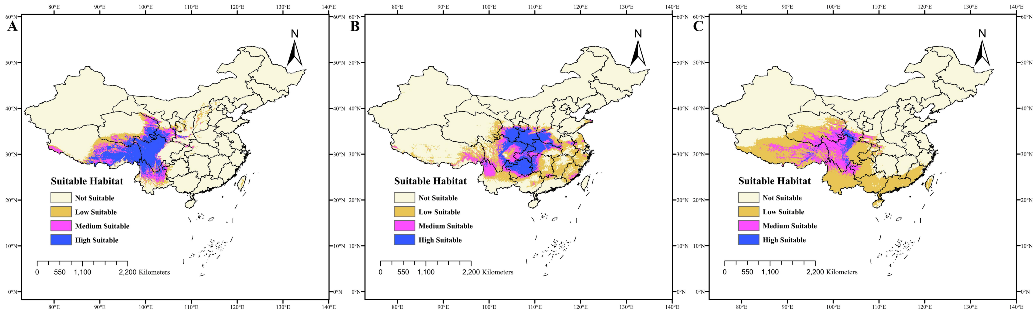

Supplementary Fig 2. The suitable habitats of three *Fritillaria* species under various scenarios in the future. (A) The suitable habitats of *F. delavayi* under SSP585 scenario at 2061-2080; (B) The suitable habitats of *F. taipaiensis* under SSP126 scenario during 2021-2040; (C) The suitable habitats of *F. wabuensis* under SSP585 scenario during 2081-2100, respectively. MaxEnt v3.3.1: https://biodiversityinformatics.amnh.org/open_source/maxent/, ArcGIS v10.2: https://www.arcgis.com/.
