# Supplementary material for: Biomod2 modeling for predicting the potential ecological distribution of three Fritillaria species under climate change
Source: Sci Rep. 2023 Nov 1;13:18801. doi: 10.1038/s41598-023-45887-6 (PMC10620159; doi:10.1038/s41598-023-45887-6)
Supplement: Supplementary file 3 — Supplementary Table 1. [file 41598_2023_45887_MOESM3_ESM.docx]

Supplementary Table 1 Environmental factor information in this study.

| Variable |  | Description | Unit |
| --- | --- | --- | --- |
| bio1 |  | Annual mean temperature | °C |
| bio2 |  | Monthly diurnal range | °C |
| bio3 |  | Isothermality (bio2/bio7 ×100) | - |
| bio4 |  | Temperature Seasonality (standard deviation×100) | - |
| bio5 |  | Max temperature of warmest month | °C |
| bio6 |  | Min temperature of coldest month | °C |
| bio7 |  | Temperature annual range (bio5-bio6) | °C |
| bio8 |  | Mean temperature of wettest quarter | °C |
| bio9 |  | Mean temperature of driest quarter | °C |
| bio10 |  | Mean temperature of warmest quarter | °C |
| bio11 |  | Mean temperature of coldest quarter | °C |
| bio12 |  | Annual precipitation | mm |
| bio13 |  | Precipitation of wettest month | mm |
| bio14 |  | Precipitation of driest month | mm |
| bio15 |  | Precipitation seasonality (Coefficient of variation) | - |
| bio16 |  | Precipitation of wettest quarter | mm |
| bio17 |  | Precipitation of driest quarter | mm |
| bio18 |  | Precipitation of the warmest quarter | mm |
| bio19 |  | Precipitation of the coldest quarter | mm |
| elev |  | altitude | m |
| aspect |  | aspect |  |
| slope |  | slope |  |
