# Supplementary material for: Biomod2 modeling for predicting the potential ecological distribution of three Fritillaria species under climate change
Source: Sci Rep. 2023 Nov 1;13:18801. doi: 10.1038/s41598-023-45887-6 (PMC10620159; doi:10.1038/s41598-023-45887-6)
Supplement: Supplementary file 8 — Supplementary Table 6. [file 41598_2023_45887_MOESM8_ESM.docx]

Supplementary Table 6. Evaluation of MaxEnt, GARP and Biomod2 models at various training/testing sets.

| Species | MaxEnt | | | GARP | | | Biomod2 | | |
| --- | --- | --- | --- | --- | --- | --- | --- | --- | --- |
|  | 85/15（training/testing） | 80/20（training/testing） | 75/25（training/testing） | 85/15（training/testing） | 80/20（training/testing） | 75/25（training/testing） | 85/15（training/testing） | 80/20（training/testing） | 75/25（training/testing） |
| *F.delavayi* | 0.974/0.166/0.298 | 0.976/0.220/0.353 | 0.973/0.131/0.236 | 0.662/0.381/0.324 | 0.676/0.402/0.351 | 0.666/0.388/0.332 | 0.999/0.964/0.970 | 0.999/0.964/0.958 | 0.995/0.941/0.946 |
| *F.taipaiensis* | 0.976/0.120/0.302 | 0.976/0.111/0.296 | 0.976/0.073/0.201 | 0.570/0.196/0.139 | 0.593/0.252/0.186 | 0.593/0.252/0.187 | 0.996/0.956/0.963 | 0.997/0.956/0.963 | 0.995/0.925/0.941 |
| *F.wabuensis* | 0.916/0.056/0.306 | 0.899/0.073/0.366 | 0.926/0.246/0.038 | 0.715/0.447/0.430 | 0.719/0.445/0.438 | 0.726/0.461/0.453 | 0.961/0.821/0.902 | 0.999/0.962/0.980 | 0.997/0.960/0.941 |

Note: The AUC/KAPPA/TSS values were separated by slashes.
