# Supplementary material for: Biomod2 modeling for predicting the potential ecological distribution of three Fritillaria species under climate change
Source: Sci Rep. 2023 Nov 1;13:18801. doi: 10.1038/s41598-023-45887-6 (PMC10620159; doi:10.1038/s41598-023-45887-6)
Supplement: Supplementary file 9 — Supplementary Table 7. [file 41598_2023_45887_MOESM9_ESM.docx]

**Supplementary Table 7**. The suitable habitats of various Fritillaria species under current and future scenarios, respectively.

| Portion of area (% ) | *Fritillaria delavayi* | | | | *Fritillaria taipaiensis* | | | | *Fritillaria wabuensis* | | | |
| --- | --- | --- | --- | --- | --- | --- | --- | --- | --- | --- | --- | --- |
|  | Not suitable | Low suitable | Moderate suitable | High suitable | Not suitable | Low suitable | Moderate suitable | High suitable | Not suitable | Low suitable | Moderate suitable | High suitable |
| Current | 82.28 | 5.10 | 3.14 | 9.48 | 76.94 | 10.96 | 5.07 | 7.03 | 79.40 | 8.22 | 5.34 | 7.05 |
| SSP126 2021-2040 | 83.54 | 4.32 | 3.30 | 8.84 | 75.78 | 11.98 | 5.44 | 6.80 | 85.77 | 5.92 | 1.45 | 6.87 |
| SSP126 2041-2060 | 83.69 | 4.76 | 3.38 | 8.16 | 76.70 | 11.51 | 5.28 | 6.51 | 84.93 | 6.02 | 1.65 | 7.40 |
| SSP126 2061-2080 | 84.28 | 4.44 | 3.51 | 7.78 | 79.81 | 9.90 | 5.06 | 5.23 | 88.60 | 7.10 | 0.78 | 3.52 |
| SSP126 2081-2100 | 84.00 | 4.57 | 3.46 | 7.97 | 78.22 | 10.45 | 5.97 | 5.35 | 89.43 | 6.76 | 0.49 | 3.32 |
| SSP245 2021-2040 | 84.39 | 4.33 | 3.02 | 8.26 | 77.91 | 10.69 | 4.81 | 6.60 | 89.08 | 6.38 | 1.07 | 3.47 |
| SSP245 2041-2060 | 83.76 | 4.71 | 3.52 | 8.01 | 76.34 | 11.88 | 5.86 | 5.93 | 88.41 | 8.77 | 0.60 | 2.22 |
| SSP245 2061-2080 | 83.54 | 4.69 | 3.92 | 7.86 | 77.05 | 11.61 | 5.96 | 5.38 | 91.36 | 6.43 | 0.18 | 2.02 |
| SSP245 2081-2100 | 83.44 | 5.16 | 3.90 | 7.50 | 78.51 | 11.00 | 6.08 | 4.41 | 75.18 | 22.87 | 1.46 | 0.49 |
| SSP370 2021-2040 | 83.45 | 4.65 | 3.25 | 8.64 | 77.36 | 11.37 | 4.96 | 6.31 | 87.13 | 5.40 | 1.40 | 6.08 |
| SSP370 2041-2060 | 83.74 | 4.43 | 3.45 | 8.39 | 78.44 | 10.59 | 5.57 | 5.40 | 93.79 | 4.63 | 0.24 | 1.34 |
| SSP370 2061-2080 | 83.49 | 4.98 | 4.09 | 7.44 | 78.27 | 11.30 | 5.76 | 4.67 | 41.67 | 54.63 | 2.31 | 1.39 |
| SSP370 2081-2100 | 83.18 | 5.34 | 4.78 | 6.70 | 79.57 | 11.26 | 5.75 | 3.43 | 23.08 | 66.35 | 8.65 | 1.92 |
| SSP585 2021-2040 | 83.30 | 4.31 | 3.39 | 8.99 | 78.93 | 9.99 | 5.36 | 5.72 | 85.98 | 8.46 | 1.06 | 4.51 |
| SSP585 2041-2060 | 83.89 | 4.87 | 3.62 | 7.62 | 77.69 | 11.36 | 5.98 | 4.96 | 93.95 | 4.54 | 0.34 | 1.18 |
| SSP585 2061-2080 | 82.78 | 4.72 | 4.57 | 7.92 | 79.67 | 11.83 | 5.17 | 3.33 | 45.04 | 48.85 | 4.58 | 1.53 |
| SSP585 2081-2100 | 82.93 | 5.13 | 4.78 | 7.16 | 78.90 | 12.25 | 5.77 | 3.08 | 19.35 | 53.23 | 22.58 | 4.84 |
